# 1,2,3,4,6-Penta-*O*-galloylglucose within Galla Chinensis Inhibits Human LDH-A and Attenuates Cell Proliferation in MDA-MB-231 Breast Cancer Cells

**DOI:** 10.1155/2015/276946

**Published:** 2015-03-30

**Authors:** Shihab Deiab, Elizabeth Mazzio, Suresh Eyunni, Oshlii McTier, Nelly Mateeva, Faisel Elshami, Karam F. A. Soliman

**Affiliations:** College of Pharmacy and Pharmaceutical Sciences, Florida A&M University, Tallahassee, FL 32307, USA

## Abstract

A characteristic feature of aggressive malignancy is the overexpression of lactic acid dehydrogenase- (LDH-) A, concomitant to pericellular accumulation of lactate. In a recent high-throughput screening, we identified *Rhus chinensis* (Mill.) gallnut (RCG) (also known as Galla Chinensis) extract as a potent (IC_50_ < 1 *µ*g/mL) inhibitor of human LDH-A (*h*LDH-A). In this study, through bioactivity guided fractionation of the crude extract, the data demonstrate that penta-1,2,3,4,6-*O*-galloyl-*β*-D-glucose (PGG) was a primary constituent responsible for *h*LDH-A inhibition, present at ~9.95 ± 0.34% dry weight. Theoretical molecular docking studies of *h*LDH-A indicate that PGG acts through competitive binding at the NADH cofactor site, effects confirmed by functional enzyme studies where the IC_50_ = 27.32 nM was reversed with increasing concentration of NADH. Moreover, we confirm protein expression of *h*LDH-A in MDA-231 human breast carcinoma cells and show that PGG was toxic (LC_50_ = 94.18 *µ*M), parallel to attenuated lactic acid production (IC_50_ = 97.81 *µ*M). In a 72-hour cell proliferation assay, PGG was found to be a potent cytostatic agent with ability to halt cell division (IC_50_ = 1.2 *µ*M) relative to paclitaxel (IC_50_ < 100 nM). In summary, these findings demonstrate that PGG is a potent *h*LDH-A inhibitor with significant capacity to halt proliferation of human breast cancer cells.

## 1. Introduction

Cancer is the second leading cause of death worldwide. While major advances have been made in design of chemotherapy agents, radiation, and surgical procedures, mortality rates remain high. Terminal cancer requires innovative therapeutic approaches, which are currently limited. Traditional use of herbs to treat diverse human illnesses dates back to thousands of years, but in today's society the use of natural product remedies is defined amongst others under the classification of “complementary and alternative medicines” (CAMs) [1–3]. The field of CAM is of global interest, in particular to augment traditional chemotherapies, prevent cancer remission, or serve as basic chemopreventive strategies [4–7].

*Rhus chinensis* (Mill.) belongs to the genus* Rhus* and the family* Anacardiaceae*.* Rhus chinensis* gall (RCG) is the abnormal growth of* Rhus chinensis* leaves caused by Chinese aphid* Schlechtendalia chinensis* [8, 9], also used for decades by indigenous people in the treatment of diarrhea, hemorrhage, and inflammation [10–17]. In a recent study conducted in our laboratory, a high-throughput screening of natural products revealed the extract of RGC to halt the proliferation of human breast cancer cells [7] and inhibit* h*LDH-A [18], an enzyme which plays a significant role in driving aggressive malignancies [2, 19–21], chemo- and radiotherapy resistance [22, 23], tumorigenic potential [24–26], and metastatic processes [22]. It is believed that the abundance of lactic acid produced and released by tumor cells assists in invasion and motility [27] where a low pH weakens the extracellular stroma which helps tumor cells to dislodge and burrow in the blood and lymphatic systems [28].

Given the potential value in the identification of novel* h*LDH-A inhibitors to augment targeted cancer therapies [16, 29], we further investigate the constituent properties of* Rhus chinensis *gallnut so as to isolate the chemical responsible for* h*LDH-A inhibition. This was achieved through bioactivity guided fractionation, theoretical molecular docking, and functional enzyme studies, followed by* in vitro* evaluation in a human breast cancer cell line with dominant protein expression of* h*LDH-A.

## 2. Materials and Methods

Materials used were Hanks Balanced Salt Solution, 4-(2-hydroxyethyl)-1-piperazineethanesulfonic acid (HEPES), ethanol, 96-well plates, general reagents and supplies, and* h*LDH-A from Abcam (Cambridge, MA, USA).* Rhus chinensis* gallnut was purchased from Kalyx Natural Marketplace (Camden, NY), cell lines were purchased from ATCC (Manassas, VA, USA), and penta-1,2,3,4,6-*O*-galloyl-*β*-d-glucose, chemical reagents, and HPLC supplies and columns were purchased from Sigma Aldrich (St. Louis, MO, USA) and VWR International (Suwanee, GA, USA).

### 2.1. Herbal Extract

Dry RCG (500 g) was ground, homogenized, and then extracted in 90% ethanol three times for 24 h and the solutions were then combined and evaporated to obtain the crude extract. Extract was then dissolved in water and successively fractionated by liquid-liquid partitioning between petroleum ether (three times, 200 mL), ethyl acetate (six times, 300 mL each), and n-butanol (three times, 200 mL each) to yield a petroleum ether soluble portion 3.5 g, ethyl acetate soluble portion 86 g, n-butanol soluble portion 7 g, and water-soluble portion 12 g. The different fractions were tested against* h*LDH-A (0.02 units/mL) activity at concentrations ranging from 0.7 to 0.0007 mg/mL (all serial dilutions were made using diluents consisting of HBSS with 10-mM HEPES adjusted to a pH 7.4). The dried ethyl acetate fraction showed* h*LDH-A inhibitory activity at a concentration of 0.0007 mg/mL.

### 2.2. Bioactivity-Guided Isolation and Identification

The ethyl acetate fraction (20 g) was flash chromatographed over silica gel (200–400 mesh) and eluted at 10 mL/min with chloroform: methanol gradient (100 : 0, 10 : 90) solvent system. A total of 500 (15 mL) fractions were collected. Fractions were pooled according to their *R*_*f*_ value on TLC and then combined to give 5 distinct fractions which were then evaporated to yield 0.5 g (1–166), 11.5 g (167–215), 4.2 g (216–267), 2.1 g (268–350), and 1.2 g (351–500). The resulting fractions were tested for activity at 0.7–0.0007 mg/mL. Fractions (167–215) showed significant* h*LDH-A inhibitory activity relative to other fractions. 1.5 g of the active fraction, 167–215, was further analyzed and separated into four major fractions by RP-HPLC with UV detection at 214 nm on PRP-15 um 15 × 2.1 mm column and eluted with a gradient system of acetonitrile: water consisting of 0.2% TFA.

Fraction 1 (0.8 g) showed significant activity compared to the other three fractions and was further separated over RP-column chromatography on a C18–125 Å 55–105 um column and eluted with 30% acetonitrile to yield three fractions. Fractions 2 (0.2 g) and 3 (0.15 g) showed similar activity and then were identified by 1H-NMR and 13C-NMR to be the same and then later combined. The substance extracted was identified as PGG (1,2,3,4,6-pentagalloylglucose) a solid, off-white powder. The melting point was determined on a Mel-Temp II apparatus (Laboratory Devices) and found to be 337°C. MW was 940.67 C_41_H_32_O_26_. 1H NMR spectra were 7.146, 7.085, 7.061, 7.012, 6.985, 6.933, 6.279, 6.258, 5.939, 5.652, 5.622, 4.878, 4.796, 4.665, 4.524, and 4.235. 13C NMR spectra were 167.02, 166.38, 166.38, 166.10, 166.00, 165.30, 145.60, 145.51, 145.34, 139.84, 139.42, 139.20, 139.08, 120.08, 119.38, 119.25, 118.75, 109.66, 94.92, 74.72, 74.13, 68.96, and 63.92.

### 2.3. Quantification of PGG

PGG concentration in whole crude ethanol RCG extract was quantified using a Shimadzu HPLC system equipped with an SPD-20A UV detector set at 280 nm, a workstation containing EZSTART version 7.4 software and an SS420X instrument interface docked to a Waters Autosampler Model 717 Plus (Shimadzu Scientific Instruments, Columbia, MD, USA; Waters, Milford, MA, USA). Mobile phase consisted of 2.5% acetic acid in 82% acetonitrile; flow rate was isocratic at 1.4 mls/minute; the column used was a 5 um 300A 4.6 × 100 mM C-18 Venusil ABS (VWR, Radnor, PA, USA). PGG external standards were prepared in mobile phase, Chinese gallnut ethanol extract was diluted in mobile phase, and injection volume was set at 25 uL.

### 2.4. Cell Culture

MDA-MB-231 (ATCC HTB-26) human breast cancer cells were obtained from ATCC (Manassas, VA). MDA-MB-231 cells were brought up in ATCC-formulated Leibovitz's L-15 medium [Catalog number 30-2008], supplemented with 10% FBS and penicillin/streptomycin (100 U/0.1 mg/mL). After confluence, the cells were subcultured and grown in DMEM containing phenol red, 10% FBS, 4 mM L-glutamine, 20 *μ*M sodium pyruvate, and penicillin/streptomycin (100 U/0.1 mg/mL). Culture conditions were maintained [37°C-5% CO_2_/atmosphere] and every 2–5 days, the media were replaced and cells subcultured. For experiments, plating media consisted of DMEM, 1% FBS (24-hour toxicity studies), or 5% FBS (72-hour proliferation studies) + penicillin/streptomycin (100 U/0.1 mg/mL), 25 mM glucose, 2 mM sodium pyruvate, and 3 mM L-glutamine.

### 2.5. Cell Count

Viable cell count was quantified using resazurin (Alamar Blue) indicator dye [30]. A working solution of resazurin was prepared in sterile PBS, phenol red (0.5 mg/mL), and added (15% v/v) to each sample. Samples were returned to the incubator for 6–8 hr, and reduction of the dye by viable cells (to resorufin, a fluorescent compound) was quantitatively analyzed using a microplate fluorometer, Model 7620, version 5.02 (Cambridge Technologies Inc., Watertown, MA) with settings at [550/580], [excitation/emission].

### 2.6. Human LDH-A Activity

A continuous* h*LDH-A assay using recombinant full length human LDHA (amino acids 1–332) with N terminal His tag, 352 amino acids with tag, and MW 38.8 kDa, enzyme commission (EC) number 1.1.1.27 (BRENDA | IUBMB) (Abcam, Cambridge, MA), was used, where we previously confirmed the identity of the enzyme using matrix assisted laser desorption ionisation (MALDI) mass spectrometry and analysis by Mascot ID [18]. Briefly, the enzyme assay buffer consisted of HBSS + calcium and Mg, pH adjusted to 7.0. PGG was added at various concentrations to* h*LDH-A enzyme (final concentration .02 units/mL) and *β*-nicotinamide adenine dinucleotide, reduced form solution (*β*-NADH) (final working concentration of 500 *μ*M), and a prereading at 340 nm was established to eliminate background. The reaction was started with a solution containing the substrate pyruvate (final concentration = 3 mM) and a reading was taken intermittently over 60 minutes at 340 nm using a 96-well microplate reader (Bio-Tek Instruments, Inc., Winooski, VT, USA).

### 2.7. Western Blot:* h*LDH-A Protein Expression

The presence of* h*LDH-A in MDA-MB-231 cells was determined using recombinant* h*LDH-A as the standard. Cells were washed and centrifuged and the supernatant was discarded using ice-cold sterile PBS at 4°C. The pellet was resuspended and homogenized/sonicated in RIPA lysis buffer containing protease inhibitors. Samples were placed on ice for 30 min and centrifuged at 10,000 ×g for 10 minutes at 4°C. The supernatant was added at 1 : 1 of Laemmli sample buffer (Biorad number 161-0737) + fresh *β*-ME and boiled for 5 minutes. Approximately 50 *μ*g of protein was loaded/lane and separated using 5%–15% SDS-PAGE gels, running buffer, 25 mM Tris, 192 mM glycine, and pH 8.3 (Biorad #161-0734) and applying 200 constant V constant ~35 min. The proteins were transferred to polyvinylidene fluoride membranes (100 V for 30–60 minutes) in ice-cold transfer buffer containing 25 mM Tris, 192 mM glycine, and 20% methanol. The membranes were placed in a blocking buffer consisting of 5% bovine serum albumin fraction V (BSA) w/v in TBS + 0.05% Tween-20, pH 7.4. The membranes were washed and placed in 1° rabbit anti-human LDH-A antibody (1 : 500) containing 1% BSA in TTBS at RT for 2 Hr. The membranes were washed in TTBS and incubated in 2° goat anti-rabbit IgG (Fc specific) peroxidase conjugate (1 : 4000) in 2% nonfat dried milk in PBS for 1.5 Hr at RT. After a final wash, peroxidase was detected with Sigma FAST DAB (3,3′-diaminobenzidine tetrahydrochloride) with a metal enhancer cobalt chloride. Images were scanned using an Epson Stylus CX-8400. Intensity analysis was performed using ImageJ software provided from the National Institutes of Health [31].

### 2.8. Lactic Acid Determination

Cellular production of lactic acid was determined in 96-well plates using a colorimetric enzymatic assay (procedure number 735, Sigma Diagnostics, St. Louis, MO). Briefly, lactate was quantified by conversion to pyruvate and H_2_O_2_ using a base lactate reagent containing lactate oxidase (400 U/L) and horseradish peroxidase 2400 U/L (Trinity Biotech Jamestown, NY, USA). The reagent was added to a chromogen and samples were incubated for 10 minutes at 37°C. Lactate was quantified at 490 nm on a UV microplate spectrophotometer (BioTek Instruments, Inc., Winooski, VT, USA).

### 2.9. Molecular Docking

The X-ray crystal structure of human LDH (M form), predominantly found in human muscle, was downloaded from the RCSB protein data bank (PDB: 1I10). Chain A was extracted and selected for the docking studies. The chain A of the protein was then refined using the structure preparation tool of the biopolymer module offered by Sybyl-X 1.3 suite [26]. In this process, mislabeled atom types from the pdb file were corrected, backbone and side chains repaired, side-chain bumps fixed, side chain amides checked to maximize potential hydrogen bonding, and all hydrogen atoms added to the protein. The protein was then subjected to energy minimization following the gradient termination of the Powell method for 10,000 iterations using MMFF94s force field incorporating MMFF94 charges with nonbonding cut-off set at 8.0 and dielectric constant set at 1.0. The resulting refined protein was used for docking purposes.

The 3D conformer of the compound in present study, PGG structure, was download from the PubChem database and subjected to minimization following the gradient termination of the conjugate gradient method for 5000 iterations using Tripos force field incorporating Gasteiger-Huckel charges with the nonbonding cut-off set at 8.0 and dielectric constant set at 1.0. Thus optimized compound PGG was docked in the catalytic active site of the chain A of the LDH protein complex (PDB: 1I10) using high precision Surlex-Dock GeomX program as implemented in Sybyl software by incremental construction approach of building the structure in the active site so as to favor the binding affinity. In this process, initially, protomol was generated using the bound ligand 1,4-dihydronicotinamide adenine dinucleotide (NAI), allowing us to map the active site for docking the test ligand PGG. During the entire docking process, ring flexibility of the ligand was considered. Predock and postdock minimizations were performed and a self-scoring term was included for optimum results.

### 2.10. Statistics

Statistical analysis was performed using Graph Pad Prism (version 3.0; Graph Pad Software Inc., San Diego, CA, USA) with IC_50_s determined by regression analysis using Origin Software (OriginLab, Northampton, MA). Significance of difference between multiple groups was assessed using a one-way ANOVA, followed by Bonferroni's multiple comparison test or Student's *t*-test.

## 3. Results

High-throughput screening has identified* Rhus chinensis* gallnut ethanol extract with inhibitory activity on human recombinant* h*LDH-A. As previously reported, this was one of the most potent natural products elucidated with an IC_50_ < 1 *μ*g/mL [18]. In order to elucidate the active constituents within the RCG extract responsible for* h*LDH-A inhibition, bioactivity guided fractionation (Figure 2(a)) was conducted to where* h*LDH-A activity was inhibited by the ethyl acetate fraction (167–215) (Figures 2(b), and 2(c)), which further yielded two active fractions within (Figure 2(d)).

Fractions 2 (0.2 g) and 3 (0.15 g) showed similar activity and then were identified by 1H-NMR and 13C-NMR to be the same and then later combined. The molecule is identified as PGG (1,2,3,4,6-pentagalloylglucose) which is a solid and an off-white substance. The melting point was determined on a Mel-Temp II apparatus (Laboratory Devices) and found to be 337°C. MW was 940.67 C_41_H_32_O_26_. 1H NMR spectra were 7.146, 7.085, 7.061, 7.012, 6.985, 6.933, 6.279, 6.258, 5.939, 5.652, 5.622, 4.878, 4.796, 4.665, 4.524, and 4.235. 13C NMR spectra were 167.02, 166.38, 166.38, 166.10, 166.00, 165.30, 145.60, 145.51, 145.34, 139.84, 139.42, 139.20, 139.08, 120.08, 119.38, 119.25, 118.75, 109.66, 94.92, 74.72, 74.13, 68.96, and 63.92. Quantification and further validation of PGG in RCG extract were accomplished with HPLC (Figure 3) where an external standard (b) versus extract (a) indicating PGG was present at 24.8 ± .85 *μ*g/mL, which is equivalent to 9.95 ± .34 of crude extract.

**Figure 1:**
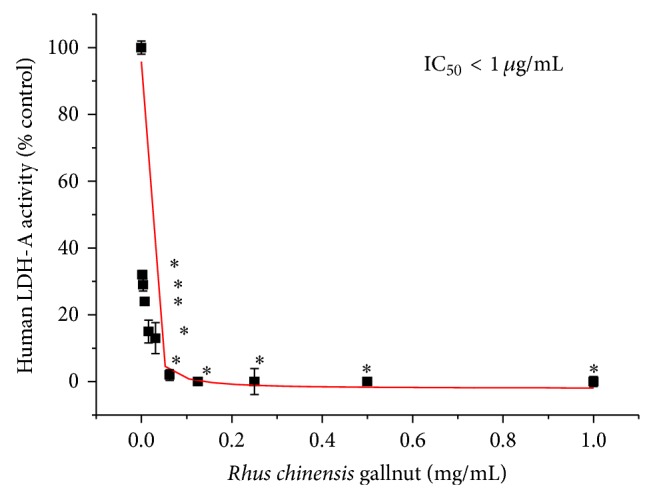
Potent* h*LDH-A enzyme inhibition by RCG extract. The data represent* h*LDH-A activity as % control (NADH oxidation) at 60 minutes and are expressed as the mean ± SEM, *n* = 4, with significance from controls using a one-way ANOVA followed by a Bonferroni's multiple comparison test; ^∗^ = *P* < 0.05, IC_50_ < 1 *μ*g/mL.

**Figure 2:**
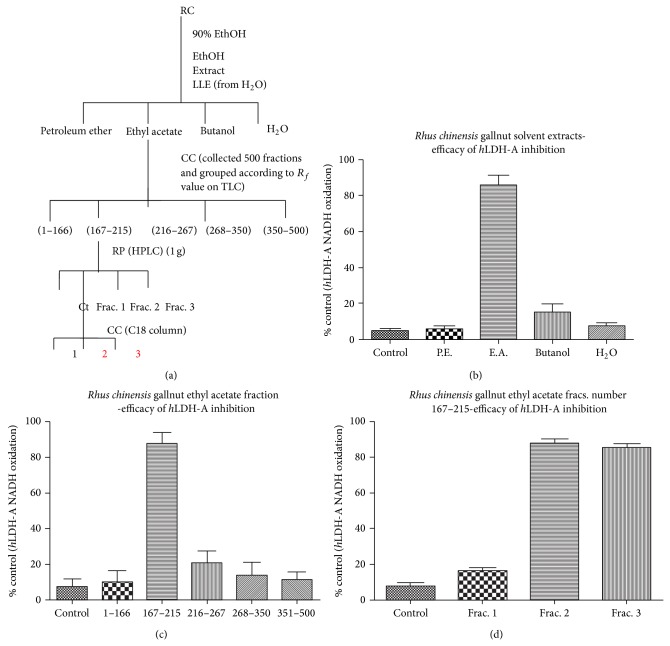
(a) Process of separation and isolation of* h*LDH-A inhibitor fractions from RCG. (b)* h*LDH-A inhibition by solvent extracts (PE: petroleum ether, EA: ethyl acetate). The data represent* h*LDH-A activity as % control and are expressed as the mean ± SEM, *n* = 4. (c)* h*LDH-A enzyme inhibition by further fractionation of the ethyl acetate fraction. The data represent* h*LDH-A activity as % control and are expressed as the mean ± SEM, *n* = 4. (d)* h*LDH-A enzyme inhibition by further fractionation of the ethyl acetate 167–215 fractions. The data represent* h*LDH-A activity as % control and are expressed as the mean ± SEM, *n* = 4.

**Figure 3:**
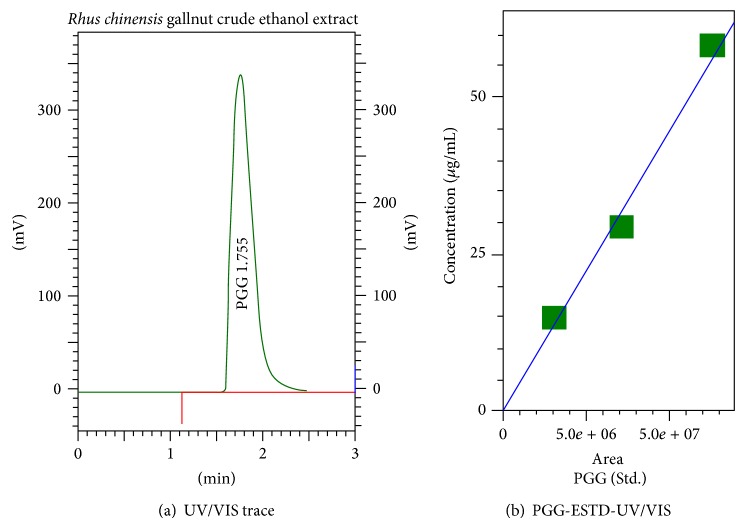
PGG concentration of RGC crude ethanol extract (a) determined by HPLC at 280 nm using external PGG standard (b). Crude extract concentration of PGG was approximately 9.95 ± .34% of weight (Chinese gallnut extract (250 *μ*g/mL) = 24.88 ± 0.85 *μ*g/mL), *n* = 4.

**Figure 4:**
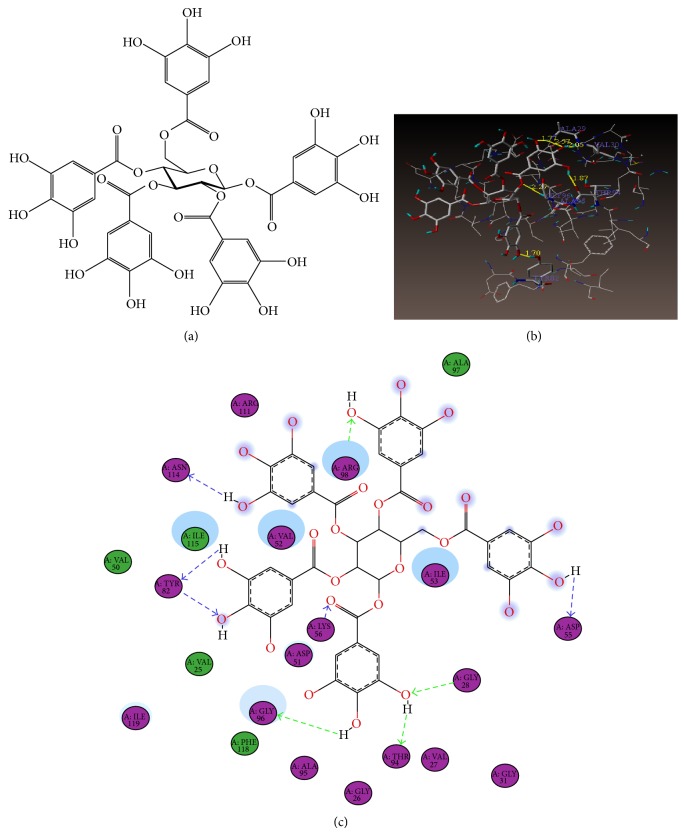
Molecular docking studies (PGG inhibition of human LDH-A). (a) Structure of 1,2,3,4,6-*O*-galloy1-*β*-D-glucose, pentagalloylglucose. (b) Binding mode of compound CID65238, PGG, in the coenzyme active site of* h*LDH-A (PDB: 1∣10). (c) Simplified binding mode of compound CID65238, PGG, in the coenzyme binding site of* h*LDH-A (PDB: 1∣10).

**Figure 5:**
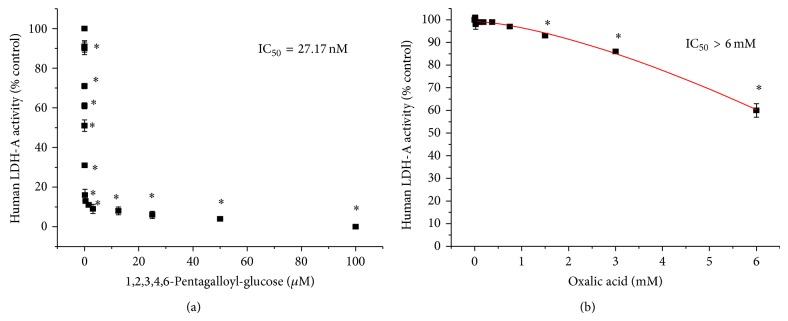
(a)* h*LDH-A (0.02 units/mL) enzyme inhibition by PGG. The data represent* h*LDH-A activity as % control (NADH oxidation) at 60 minutes and are expressed as the mean ± SEM, with significance from controls determined with a one-way ANOVA followed by Bonferroni's multiple comparison test; ^∗^ = *P* < 0.05, *n* = 4. IC_50_ = 27.17 nM. (b)* h*LDH-A enzyme inhibition by reference known inhibitor (oxalic acid). The data represent* h*LDH-A activity as % control (NADH oxidation) at 60 minutes and are expressed as the mean ± SEM, with significance from controls determined using a one-way ANOVA followed by Bonferroni's multiple comparison test; ^∗^ = *P* < 0.05, *n* = 4, IC_50_ > 6 mM.

**Figure 6:**
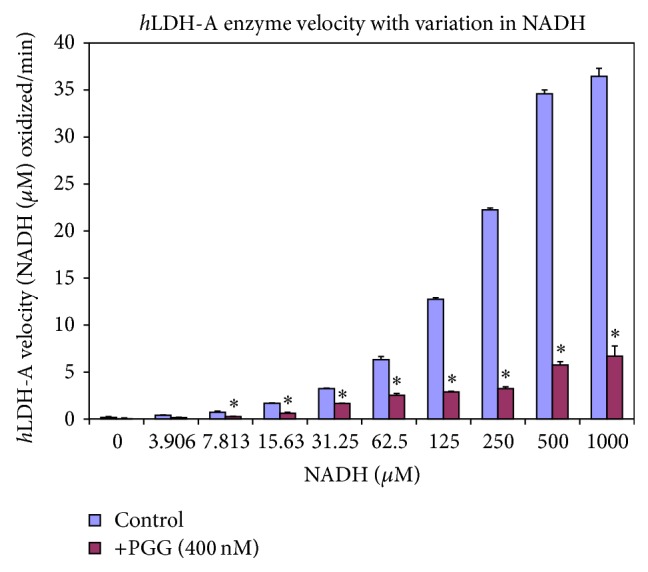
*h*LDH-A enzyme velocity ± 400 nM PGG. The data are presented as the average rate of reaction: NADH oxidized over 60 minutes at RT and displayed as the mean ± SEM, *n* = 4. Significance of difference between NADH controls and PGG treated samples was analyzed by a Student's* t*-test; ^∗^ = *P* < 0.05.

To assess if PGG could theoretically inhibit* h*LDH-A, molecular docking studies were conducted and the data show predicted competitive binding of PGG (Figure 4(a)) within the NADH binding cofactor site of the* h*LDH-A enzyme (Figures 4(b) and 4(c)). Docking studies showed H bond interactions between the docked ligand PGG and the protein in the cofactor site (Figures 5(a) and 5(b)). HO⋯HO (TYR_82) = 1.70 Å; HO⋯HN (ALA_29) = 1.77 Å; OH⋯O=C (THR_94) = 1.87 Å; HO⋯HN (VAL_30) = 2.05 Å; HO⋯HN (GLY_96) = 2.27 Å; HO⋯HN (ALA_29) = 2.57 (Figures 1 and 2). When compound PGG was docked alongside the bound ligand 1,4-dihydronicotinamide adenine dinucleotide (NAI), the total scores which are expressed as—log(*K*_*d*_) obtained were 5.86 and 12.61, respectively, for PGG and the bound ligand NIA, respectively. These effects were also confirmed by functional studies (Figure 5(a)), where PGG was found to be an extremely potent* h*LDH-A inhibitor within the therapeutic range IC_50_ = 27.17 nM compared to a known* h*LDH-A inhibitor oxalic acid (Figure 5(b)) demonstrating a relative weak potency reflected by an IC_50_ > 6 mM. Competitive binding of PGG versus NADH clearly shows that the site has greater affinity for PGG than NADH (Figure 6).

**Figure 7:**
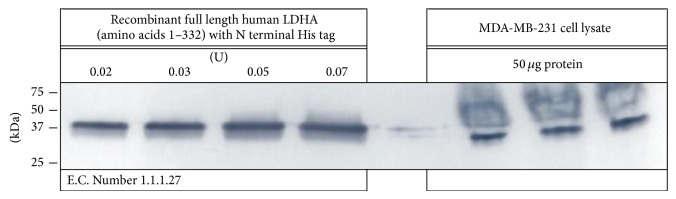
Western blot confirmation of human* h*LDH-A in MDA-MB-231 breast tumor cells (*in vitro *studies) and pure recombinant* h*LDH-A used (enzyme kinetic studies).

In order to determine the effects of PGG* in vitro*, a human breast cancer cell line (MDA-MB-231) lysate was evaluated for the baseline expression of* h*LDH-A by Western blot (Figure 7). These findings validate the identity of the recombinant protein used in this study, as well as demonstrating dominant expression level in MDA-MB-231 cells, further subject to* in vitro* effects incurred by PGG.

**Figure 8:**
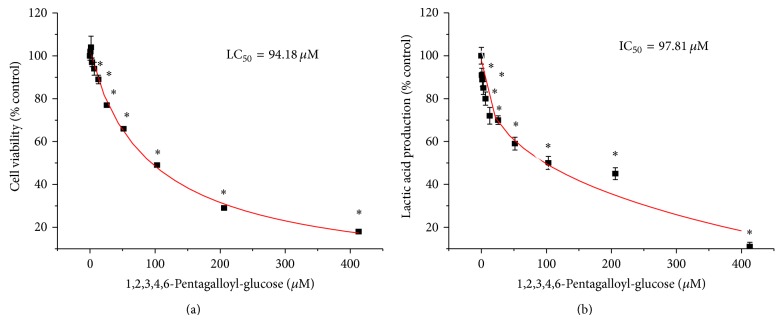
The effect of PGG on viability/metabolism and lactic acid produced by MDA-MB-231 human breast cancer cells (5.0 × 10^6^ cells per well) low serum [2%] phenol-free media supplemented with amino acids at 24 hours. The data represent cell viability (% control) (a) and lactic acid (% control) (b) and are expressed as the mean ± SEM with significance from controls determined using a one-way ANOVA followed by Bonferroni's multiple comparison test; ^∗^ = *P* < 0.05, *n* = 4.

**Figure 9:**
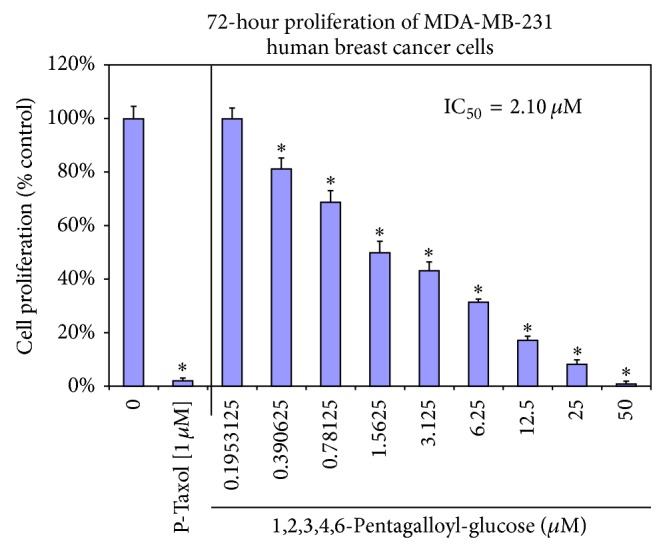
Inhibitory growth properties of PGG on MDA-MB-231 human breast carcinoma cells relative to 1 *μ*M paclitaxel. The data represent growth from an initial plating density of 4,000 cells/well in 96-well plates over a 72-hour period as proliferation (% untreated controls) established with resazurin (Alamar Blue) fluorometric analysis. The data represents cell proliferation as % control and is presented as the mean ± SEM, *n* = 4, with significance from controls determined using a one-way ANOVA followed by Bonferroni's multiple comparison test; ^∗^ = *P* < 0.05.

*In vitro* analyses on the lethality of PGG versus reduction in lactic acid accumulated in the supernatant were near identical (Figures 8(a) and 8(b)). However, given that all toxic compounds regardless of mechanism of action will reduce lactic acid as a process of cell death in tumor cells, there is no conclusion drawn from this data. If loss of lactate significantly preceded cell death, one could say there was a correlation, but further analysis will be required. The antiproliferative effects of PGG relative to paclitaxel were evaluated over a three-day growth period (Figure 9). In this study, we provide data to support that PGG, a component of RGC extract, can inhibit human LDH-A and halt proliferation of a human tumor cell line MDA-MB-231, which highly expresses the human LDH-A protein.

## 4. Discussion

The data in this study confirm penta-1,2,3,4,6-*O*-galloyl-*β*-d-glucose (PGG)—a component in RCG—as a newly identified* h*LDH-A inhibitor with potential application as a CAM for use in cancer treatment. Our work supports/builds on the findings of others in describing therapeutic value of this molecule as an anticancer agent, now having been established in the suppression of prostate cancer metastasis [32], angiogenesis, with capacity to initiate apoptosis [33–36], and halt cell cycle at the G1 phase [37, 38] at the DNA replication S-phase [39], corresponding to the primary basis of proposed therapeutic use in treatment of cancers [40].

At the same time, the RGC extract as a whole is equally capable of inhibiting angiogenesis, inducing apoptosis in cancer cells [34] and as a cytostatic agent [7]. Moreover, its medicinal value has been described in the scientific literature for its multidimensional therapeutic value, also effective against harmful intestinal and periodontal bacteria [9, 41], hepatitis carcinoma virus [15], severe acute respiratory syndrome corona [42] and as an anti-inflammatory [11, 43–45].

The data in this study also confirm the ability of PGG to inhibit* h*LDH-A which is a therapeutic target in particular given its role in tumor initiation, progression, and metastasis [46, 47] and in aggressive malignancies such as pheochromocytomas, paragangliomas [48], or breast cancer [49]. Interestingly, while LDH is a specific therapeutic target in tumor cells, its exact mechanism of action to exert anticancer effects remains unknown.

It is believe that elevated* h*LDH-A protein expression in tumor cells, and subsequent over production of lactic acid contributes to development of radiation and chemotherapy resistance [27, 28]. Tumor cell overproduction of lactic acid (in the absence or presence of O_2_) was described almost a century ago by Otto Warburg, having been termed “the Warburg effect.” While many scientists theorize that this aberration is a function of altered metabolism, studies in our laboratory exploring this phenomenon using current technologies such as whole-genomic, proteomic MALDI-TOF-MS and metabolite analysis show that the Warburg effect has a functional role not in metabolism, but in regulating acidic pericellular pH (pHe). For some unknown reason, cancer cells inherently thrive in slightly acidic pH, are extremely vulnerable to necrosis as the pH shifts toward alkaline, and will sustain a steady state pH through metabolic inversion or fluctuating dominance between glycolytic-rate substrate level phosphorylation (SLP) and mitochondrial (mt) oxidative phosphorylation to control lactic acid production [50]. If this is the case, then* h*LDH-A would comprise a vehicle to maintain acidic pH where tumor cells thrive, but the question after much work still remains as to why tumor cells require ample concentrations of acid.

Acidity alone, can trigger aggressive forms of malignancy, augment metastases, and initiate chemoresistance all of which correlate to low survival rates [51, 52]. Data collected from our lab and by others seems to indicate that acid can turn on anabolic processes in tumor cells, due to energy efficiency with elevated protein expression for nutrient sensor G*β*L (G protein, beta protein subunit-like), a component of* m*TOR (mammalian target of rapamycin), PI3K/Akt signaling, and its downstream eIF4E tumor promoting target [53]. These changes are indicative that feedback sensors (such as acid) could switch the metabolic state of tumor cells from anabolic to catabolic [54].

While the exact role of* h*LDH-A remains elusive, it is known that* h*LDH-A knockdown, or lowering the functional capacity of* h*LDH-A, can lead to suppressed tumor growth and metastasis indicating this enzyme as a novel targeted cancer therapy strategy. In this study, we provide data to support therapeutic efficacy of PGG, a component of RGC extract, to inhibit human LDH-A and halt proliferation of a human tumor cell line MDA-MB-231, which highly expresses the* h*LDH-A protein.

## 5. Conclusion

Pentagalloylglucose, PGG, a component of RGC, shows a remarkable competitive inhibitory activity against* h*LDH-A at the NADH cofactor docking site. Its effect in tumor cells baring high expression of* h*LDH-A resulted in blocking proliferation. Future research will be required to determine in detail cause-effect relationship between* h*LDH-A inhibition and biochemical metabolic or process related effects in tumor cells.
